# The New Era of Veterinary Medicine1President’s Address delivered before the New York State Veterinary Medical Society, Brooklyn, New York, September 9, 1902.

**Published:** 1902-11

**Authors:** James Law

**Affiliations:** Ithaca, N. Y.


### THE NEW ERA OF VETERINARY MEDICINE.[1]

By Prof. James Law, F.R.C.V.S.,
ITHACA, N. Y.

(Concluded from page 612.)

Let us now glance for a moment at tetanus. Until comparatively recently no one scarcely suspected that tetanus was an infectious disease. It was believed to be the result of local irritation and nervous excitement, and there is yet a widespread idea that any violent pain is likely to bring on the affection. This received countenance from the frequency with which it followed deep wounds of the palms and soles in man, the feet, tail, and testicles in the horse, from lesions of unyielding fibrous tissues, and from gunshot and bayonet injuries. The fact was overlooked that the affection often started during or after cicatrization, when the real irritation had in a great measure passed. Since the discovery of the bacillus of Nicolaier we know that this anaërobic germ flourishes in deep, closed wounds only, under the skin, fascia, or tendon ; therefore, in stab and bullet traumas par excellence, in which there is abundant exudate outside all circulation of blood or oxygen, and that it will not invade the circulating blood in which oxygen is relatively abundant. We are no longer in the dark, left to meet it blindly by antispasmodics alone, but we know how to prevent it by asepsis and antisepsis of wounds, by exposing deep dirty wounds to the action of the air, by dressing with antiseptics the navel of the newborn, and by ridding the subject of intestinal worms which make infection atria for the bacillus.

[1] President's Address delivered before the New York State Veterinary Medical Society, Brooklyn, New York, September 9, 1902.

Formerly a large number of young animals died of purulent arthritis and diarrhœal diseases very shortly after birth, the affections being attributed to weakness of constitution and faulty feeding. To-day we place the finger on the source of trouble in the infective omphalitis, from which the septic microbes find their way in the blood to produce inflammatory changes, microbic degenerations, and secondary abscesses in the liver, lungs, joints, and alimentary canal.

Our herds of horses and cattle alike, and our flocks, suffered largely from abortions, which were attributed to constitutional infirmities, faulty feeding, mechanical injuries, and emotional disorders. Now we know that different infectious microbes living in the genital passages are largely the cause of the trouble, and that the malady is just as amenable to treatment and prevention by sanitary precautionary measures as are any of the diseases that have been long recognized as due to contagion.

Many of us can recall when actinomycosis was known as cancer, clyers, spina ventosa, or osteosarcoma, terms that served to cloak our ignorance of its real cause and pathology. To-day we know that it is simply a culture in the living tissues of the beautiful ray fungus or actinomycosis; we can trace its secondary causes in the sowing of its seed in wounds and natural openings and recesses, and can not only ward off its approaches, but in all moderate cases we can subject it to effective treatment, medical and, it may be, surgical as well.

Dourine has been known in Europe since 1796, and in 1882 was introduced into Illinois in the body of a French horse. Until very recently, however, it was very much misunderstood. Bouley, Trasbot, and others accepted the fiction that it is syphilis communicated from man to horse, and propagated thereafter as an equine disease. Others considered it as glanders affecting the genital and nervous systems. To-day we recognize its cause in the trypanosoma equiperdum, an infusorial parasite which lives in the blood and genital passages, and can be successfully transferred to the dog. Surra, or rot of the hot rainy season in Hindustan and neighboring countries, like rinderpest, now threatens us through our Philippine dependencies.

This affection of solipeds—camels and elephants—was supposed to be a form of remittent or bilious fever allied to that of man, but is now traced to another trypanosoma (T. Evansu), differing from that of dourine, among other things in that it is transferred from animal to animal by blood-sucking flies and other insects.

The nagana of East Africa, due to another trypanosoma (T. Brucei), and which is not infecting to elephants, was only known late in the last century, and was long attributed to a special poison instilled into the wound by the tsetse fly (Glossina Morsitans), which is now known to convey the trypanosoma.

Here we have a new class of infectious diseases introduced into our veterinary literature, one of which (dourine) has already gained a foothold in the United States, and a second (surra) threatens us through our Eastern dependency. It may be but the turning of a new leaf, as other animals suffer habitually in certain countries from trypanosoma; a parasite of this kind (T. Lewisi) is common in rats in Asia and Europe; another invades the systems of the dog in South Africa and Europe, and certain inscrutable diseases of South America and elsewhere are quite suggestive of this class of parasite.

In 1888 Starcovici found pyriform organisms in the blood globules of Roumanian cattle the victims of hæmoglobinuria; the following year what appeared to be the same parasite was found by Th. Smith in the blood globules of cattle suffering from the corresponding disease in America—Texas fever. Later it was found in Australian victims, and still later in the tristiza of South America. Thus was another of the most deadly infections of animals traced to its true source. But the advance of knowledge did not end here; shrewd observers as early as 1868 had noticed that Texas fever never occurred in the absence of the cattle tick, and in 1889 our colleague, Dr. F. L. Kilborn, was so impressed with this fact that he secured permission to put it to the test under the auspices of the Bureau of Animal Industry. His expectations were confirmed in every particular, and his results were soon endorsed by observations in all parts of the world in which this disease was known to prevail. To-day the bearing of the piroplasma by the tick is, perhaps, the most important fact in connection with the disease; it is the pivot upon which prevention revolves, and it bids fair to be the foundation on which the final extinction of the malady will be based.

The doctrine of tick causation has borne fruit in other directions. The louping illness of sheep in Northern Europe has been traced to the ticks in the pastures, and what was long a mystery to flock-master and veterinarian alike has been now cleared up and a substantial basis for prevention furnished. The agency of flies and other insects in the transmission of chicken cholera, typhoid fever, anthrax and other diseases in summer has been demonstrated, and certainly has taken the place of speculation in matters essential to sanitary policy.

In the same way at every step we have learned of the agency of birds, wild and tame, in the transmission of infection, sometimes as being themselves the victims of the pathogenic germs, sometimes when they become mere bearers from feeding in the same troughs with the sick animals, and other times still because they are devourers of carrion, and feed upon the carcasses of the victims of a pestilence. In their turn the vermin which approach and feed in our stables, stock yards, and parks, have to be taken into account as infection-bearers. Thus, the field of the veterinarian has been continually widening. An acquaintance with the infinitesimally small organisms in both the animal and vegetable kingdoms, and as far as possible with their life-history, become a *sine qua non* of modern practice.

In this microbian pathology is involved the whole subject of immunization and serum therapy. Into this fascinating subject I cannot enter here further than to say that immunization is mainly based on our power of investing the leucocytes and tissue nuclei with the habit of producing defensive products which are inimical to the invading microbes or chemically neutralize their toxins, while serum therapy utilizes the same antitoxins already formed, and while introduced into the system in full doses will operate at once without waiting for the work of the leucocytes in forming these defensive serums. In their action the two differ essentially in this respect, that when the leucocytes have, by contact with a non-fatal dose or doses of the toxic matters of the microbes, been habituated to the production of the requisite amount of antitoxins, the habit remains, and for a length of time the animal is immune from the effect of any ordinary dose of the microbe. When, on the other hand, the antitoxins alone are introduced into the system, the microbes and their toxins are neutralized and the animal protected from the commencing or pending attack, but the leucocytes are not stimulated to produce their own antitoxins, and the moment the defensive injection has been used up, any toxins that may be present can resume their sway.

The adaptability of these systems varies greatly in the case of different microbes and subjects. In one disease the new direction given to work off the leucocytes has an effect as lasting as life; in another, a second attack may follow in a very short time. The first is a fit subject of immunization; the second is not. Again, in one disease the microbe can be robbed of its vitality or virulence by different agencies, which do not destroy the toxins; in another the toxins are more readily destroyed than the microbe, so that the training of the leucocytes to the defensive habit becomes impossible by the same method. The principle is largely the same for all, but the *modus operandi* must vary with the disease and its product. There is no royal road to immunization or to serum therapy, but working from the same fundamental principles the technique must be worked out independently for each malady. Yet, taken as a whole, it is a division of the new medicine and one of the most fruitful branches of the microbian pathology.

But we must not confine our attention to the infinitesimal, under the delusion that here only our new pathology bears sway. We must become familiar with the animal kingdom in order to an intelligent management and control of infections that are transmitted through the instrumentality of the feral fauna.

Again, the importance of the animal kingdom has always been recognized in connection with parasitism, but in the past half century the comparatively few parasites previously esteemed important in their pathogenesis have been multiplied a hundredfold, and here again a practically new field has been opened up for veterinary study. Some of these parasites are among the most redoubtable and destructive known. Need I name, for I must do no more, the actinomyces, coccidia, strongyles of the lungs and bowels in different animals, the tænia, the bothriocephali, the sclerostomata filaria, and other blood parasites, the œsophagastomata, the trichinæ, the aspergilli, and the acarini, of internal organs in all their species and habitats. But I must not delay on the almost endless list of parasites, even to enumerate their genera.

One disease, however, has been so far omitted that it would be almost criminal to leave it out of our list. When, in 1865, Villemin published the results of his experiments in the production of tuberculosis by inoculation with the virulent matter of the tubercle it was met by an all but universal opposition. A vulgar opinion in certain localities, and a silent conviction of certain veterinarians, based upon their observations in herds almost alone, coincided with the brilliant French investigator. But this came in an age when authority could no longer crush facts, and slowly but surely the facts triumphed, and a foundation was laid for—what must still after thirty-seven years be referred to as—the *future extinction* of the most insidious and deadly plague of man and animal. Seventeen years passed ere Koch crowned the work of Villemin by the discovery of the bacillus tuberculosis, for which the world was now in a measure prepared, and now after twenty years more we are still debating the unity or plurality of the tubercle bacillus. Truth travels slowly, but under our modern *a posteriori*, or scientific method, its final triumph cannot be doubted. Already we have learned some of the variations of the dread bacillus, its lines of easy diffusion and those that prove difficult and uncertain. And already we see that varieties of this bacillus which have, in accordance with their environment, assumed habits the most diverse, can be restored to one common character even as regards pathogenesis. The stumbling blocks are being gradually removed, and we are daily approaching nearer to the time, when, for both man and beast, tuberculosis shall be shorn of its greatest dangers, and the tribute of one-eighth of humanity and a large proportion of our live-stock to the insatiable bacillus shall be reckoned a thing of the past.

Gentlemen, I have held your attention too long. I have sought to establish the position that we meet under different auspices than confronted our predecessors. Times have changed, veterinary medicine has changed, and no less our duties have changed. I have hardly touched more than one phase of the mighty transformations of the medicine of the past half century—the new microbian pathology. I would like to add that this pathology and this change are by no means limited to the field of animal plagues, but must be dealt with in the different phases and stages of the majority of what are still to be considered as sporadic diseases. And if we could enter into the allied field of medicine we would everywhere meet with changes, advances, and demonstrations only less than we find in pathogenic microbiology.

The change in conditions demand a change in our attitude and effort; as a profession we can only hope to retain the confidence and support of the public by showing that we are in full relation and sympathy with the medicine of modern times.

In the past the veterinary profession of New York have had the humiliation of seeing medical men and laymen appointed to do work which rightly belonged to the veterinarian, on the alleged ground that the latter is deficient in the education and skill necessary to fulfil the duties of the place. The profession and the public have alike suffered, and they will continue to suffer more or less until we can confute our detractors by pointing to a body of men who are amply trained for this work and whose fitness is guaranteed by the legal demand of a high standard for entering and practising the profession. We have, to a large extent, secured such a standard, one that aims higher than that of any other State in the Union. The main drawback is the illegal entry or practice in the State of those who are not up to the standard required, and who seek to secure the esteem, the confidence, and all the rights and immunities of those who have entered on practice through the legal doorway. These men are violating the law, they are doing violence to every sense of honor, and they are doing their best to degrade the veterinary profession, for by unchallenged practice in the State they are setting a standard in the mind of the stock-owner by which the whole veterinary profession is of a necessity graded.

By the State law this Society is set as a guardian over this matter. The Society must nominate the men who shall preside over the examinations for *license to practise* in New York, and on this Society is imposed the duty of authorizing the prosecution of any one who may practice without this license.

This duty presses on every veterinarian in the State. If he declines to assume it he is unfaithful to his profession; he is contributing his influence toward excluding the profession from its rightful field of work and toward the denial of the State and its great live-stock industry of the valuable service, which, under the statute, they have a right to expect. The modern advances in veterinary medicine demand of us new duties, and by the terms of the statute the responsibilities are laid upon our shoulders. The question comes to every member of this Society and to every legal veterinary practitioner in the State: Can you be trusted to meet the responsibility laid upon you? Will you prove yourselves worthy of the highly honorable duties imposed upon you by the law of the Commonwealth?

It is to be hoped that before it separates this Society will take decided steps toward the maintenance of the law in its integrity. It is to be further hoped that every member of the Society and every legal practitioner in the State will frown upon and give his active opposition to any attempt that may be made to make any man a legal practitioner by special enactment and in defiance of the existing law and of the Regents Board of Veterinary Examiners.

It need only be added that for the meeting now in session the programme presents a varied list of practical papers and surgical demonstrations which should call forth the best discussion. While we are all grateful to the gentlemen whose names are on the programme, their praiseworthy efforts will be largely lost if they fail to draw out the combined wisdom and experience of the hearers as well.

